# Signaling underlying kappa opioid receptor-mediated behaviors in rodents

**DOI:** 10.3389/fnins.2022.964724

**Published:** 2022-11-03

**Authors:** Lee-Yuan Liu-Chen, Peng Huang

**Affiliations:** Center for Substance Abuse Research, Department of Neural Sciences, Lewis Katz School of Medicine, Temple University, Philadelphia, PA, United States

**Keywords:** kappa opioid receptor, receptor phosphorylation, receptor internalization, conditioned place aversion, sedation, motor incoordination, analgesic effect, antipruritic effect

## Abstract

Kappa opioid receptor (KOR) agonists are potentially useful as analgesic and anti-pruritic agents, for prevention and treatment of substance use disorders, and for treatment of demyelinating diseases. However, side effects of KOR agonists, including psychotomimesis, dysphoria, and sedation, have caused early termination of clinical trials. Understanding the signaling mechanisms underlying the beneficial therapeutic effects and the adverse side effects may help in the development of KOR agonist compounds. In this review, we summarize the current knowledge in this regard in five sections. First, studies conducted on mutant mouse lines (GRK3-/-, p38alpha MAPK-/-, β-arrestin2-/-, phosphorylation-deficient KOR) are summarized. In addition, the abilities of four distinct KOR agonists, which have analgesic and anti-pruritic effects with different side effect profiles, to cause KOR phosphorylation are discussed. Second, investigations on the KOR agonist nalfurafine, both *in vitro* and *in vivo* are reviewed. Nalfurafine was the first KOR full agonist approved for clinical use and in the therapeutic dose range it did not produce significant side effects associated with typical KOR agonists. Third, large-scale high-throughput phosphoproteomic studies without *a priori* hypotheses are described. These studies have revealed that KOR-mediated side effects are associated with many signaling pathways. Fourth, several novel G protein-biased KOR agonists that have been characterized for *in vitro* biochemical properties and agonist biases and *in vivo* behavior effects are described. Lastly, possible mechanisms underlying KOR-mediated CPA, hypolocomotion and motor incoordination are discussed. Overall, it is agreed upon that the analgesic and anti-pruritic effects of KOR agonists are mediated via G protein signaling. However, there is no consensus on the mechanisms underlying their side effects. GRK3, p38 MAPK, β-arrestin2, mTOR pathway, CB1 cannabinoid receptor and protein kinase C have been implicated in one side effect or another. For drug discovery, after initial *in vitro* characterization, *in vivo* pharmacological characterizations in various behavior tests are still the most crucial steps and dose separation between beneficial therapeutic effects and adverse side effects are the critical determinant for the compounds to be moved forward for clinical development.

## 1. Introduction

The kappa opioid receptor (KOR), one of the three opioid receptors, belongs to the rhodopsin subclass of the G protein-coupled receptor (GPCR) superfamily. The KOR is coupled to Gi/o trimeric G proteins. Activation of the KOR by a prototypic agonist initiates G protein-mediated signaling and recruits G protein-coupled receptor kinases (GRKs) to promote phosphorylation of the KOR, which triggers β-arrestins-dependent signaling as well as regulation of the KOR, such as desensitization, internalization, and downregulation. When activated, Gα and Gβγ subunits initiated signaling, which includes modulation of adenylate cyclases, activation of G protein-activated inward-rectifying potassium (GIRK) channels, inhibition of calcium channels, enhanced phosphorylation of p42/44 mitogen-activated protein (MAP) kinase (early phase) and activation of phosphoinositide 3 kinase. β-arrestins recruited by activated KOR enhanced p38 MAP kinase phosphorylation, p42/44 MAP kinase phosphorylation (late phase) and c-Jun N-terminal kinase (JNK) phosphorylation [see (Brust, 2022) for a review and references therein].

*In vivo* KOR agonists produce analgesic and anti-pruritic responses and water diuresis (Von Voigtlander et al., 1983; Cowan and Gmerek, 1986; Simonin et al., 1998). KOR agonists may also be useful for treatment and prevention of opioid or cocaine use disorders (Simonson et al., 2015; Townsend et al., 2017; Kivell et al., 2018; Zamarripa et al., 2020b,^2021^) and for treatment of demyelinating diseases (Du et al., 2016; Denny et al., 2021; Dworsky-Fried et al., 2021; Paton et al., 2021). However, they also produce some undesired effects, such as psychotomimetic effects, dysphoria, sedation, and motor incoordination (Pfeiffer et al., 1986; Pande et al., 1996). These side effects hinder the development of KOR agonists for clinical use, except nalfurafine (formerly named TRK-820) in Japan and Korea (Kumagai et al., 2012; Kozono et al., 2018) and recently difelikefalin (previously named CR-845) in the US (Fishbane et al., 2020)^1^. Nalfurafine was approved in Japan for treatment of systemic itch associated with kidney dialysis and chronic liver diseases (Kozono et al., 2018; reviewed in Miyamoto et al., 2022). It was also approved in Korea for hemodialysis-related pruritus (Kozono et al., 2018); however, it was not approved by European Medical Agency for the same indication due to lack of efficacy. At the therapeutic anti-pruritic dose range, nalfurafine did not cause psychotomimetic effect, dysphoria or sedation, although it does get into the brain (Kozono et al., 2018). Difelikefalin, a peripherally acting KOR agonist, was recently approved by the US FDA for hemodialysis-related pruritus (Fishbane et al., 2020)^1^.

Biased agonism or functional selectivity is a popular concept in GPCR signaling (Whalen et al., 2011; Violin et al., 2014; Rankovic et al., 2016). According to this concept, an agonist may preferentially activate G protein- or β-arrestins-mediated signaling pathway.

Early reports by Bohn et al. showed that β-arrestin2 (β-arr2) deletion in mice enhanced analgesic effects of morphine and blocked tolerance to morphine-induced analgesic effects but did not affect morphine dependence (Bohn et al., 1999, 2000), consistent with the role of β-arr2 in desensitization, internalization and downregulation of the mu opioid receptor (MOR). β-arr2 deletion attenuated morphine-induced respiratory depression and inhibition of gastrointestinal transit (Raehal et al., 2005), contrary to the anticipated results if β-arr2 simply plays a role in regulating MOR activity. These observations were interpreted to mean that β-arr2 is involved in signaling leading to MOR-mediated respiratory depression and inhibition of gastrointestinal transit. These findings led to the concept of involvement of β-arr2 in MOR signaling and behavior responses and prompted the synthesis and characterization of G protein-biased MOR agonists, which presumably would cause lower degrees of respiratory depression and constipation.

Subsequently, β-arr2-mediated signaling was demonstrated for many GPCRs and G protein- and β-arr-mediated signaling are shown to result in different behaviors *in vivo*. Studies have been conducted to determine the roles of G protein and β-arrestin signaling in KOR-mediated behaviors, early on largely from Chavkin’s lab. Chavkin coworkers demonstrated that KOR agonist-induced conditioned place aversion (CPA), an animal model of dysphoria, was mediated by G-protein-coupled receptor kinase 3 (GRK3) and p38 MAP kinase, whereas KOR-mediated analgesic effects were G protein-mediated (Bruchas et al., 2006, 2007, 2011; Land et al., 2009; Bruchas and Chavkin, 2010; Ehrich et al., 2015). These findings prompted several groups to synthesize and characterize novel G protein-biased KOR agonists (see section “5. Novel G protein-biased kappa opioid receptor agonists”).

Some recent findings have challenged the concept that β-arrestins are involved in MOR-mediated behaviors. TRV130, a moderately G protein-biased MOR agonist, was found in rodents to have enhanced analgesic effects, lower tolerance and reduced respiratory depression and constipation (DeWire et al., 2013; Soergel et al., 2014). However, in clinical trials, TRV130 (oliceridine), producing equally effective analgesia as morphine, exhibited no significant difference in the incidence of respiratory depression from smorphine (Tan and Habib, 2021). In addition, Kliewer et al. (2020) reported that β-arrestin2 deletion in mice did not affect respiratory depression and constipation induced by morphine or fentanyl. The differences between the studies of Raehal et al. (2005) and Kliewer et al. (2020) may be in part due to differences in genetic background of the mice. Raehal et al. (2005) used mice of mixed C57BL/6 × 129 background, whereas Kliewer et al. (2020) used mice of C57BL/6 genetic background. In addition, mice expressing a phosphorylation-deficient MOR (i.e., G protein-biased MOR) displayed enhanced morphine analgesia and diminished morphine tolerance but unaltered or even heightened respiratory depression, constipation, and opioid withdrawal signs. These observations indicate that β-arrestin recruitment does not contribute to the severity of opioid side effects, hence, predicting that G-protein-biased MOR agonists are still likely to lead to severe adverse effects (Kliewer et al., 2019). After systemic examination of effects of G protein-biased MOR agonists (oliceridine, PZM21, and SR-17018) on several signaling endpoints not confounded by limited signal windows, Gillis et al. (2020) concluded that these agonists had low intrinsic activities at the G protein pathway, which accounted for their improved side effect profiles.

Recent trends in the development of KOR agonists for therapeutic uses include G protein-biased agonists (see section “5. Novel G protein-biased kappa opioid receptor agonists”), peripherally acting agonists [e.g., difelikefalin (CR845)] and selective KOR partial agonists [e.g., [Amo2]EM1 (De Marco et al., 2018)].

Herein we review the literature on the roles of G proteins and arrestins in KOR-mediated behaviors. In addition, functional selectivity and pharmacological characterization of nalfurafine, until recently the only KOR agonist in clinical use, are described. Moreover, large-scale high-throughput phosphoproteomic studies on mouse brains to delineate signaling mechanisms underlying KOR-mediated adverse effects are discussed. Furthermore, *in vitro* and *in vivo* characterization of novel G protein-biased KOR agonists are briefly discussed. Readers are referred to a recent comprehensive review on G protein-biased KOR agonists (French and Van Rijn, 2022). Additionally, possible mechanisms underlying KOR-mediated CPA, hypolocomotion and motor incoordination are discussed. Lastly, considerations on behavioral tests in experimental animals to better capture the human responses to KOR agonists are included. The content will be focused on KOR agonists that have been shown to have analgesic and anti-pruritic effects. Those that were studied for other effects, such as on substance abuse (Simonson et al., 2015; Townsend et al., 2017; Kivell et al., 2018; Zamarripa et al., 2020a,b) and on remyelination (Du et al., 2016; Denny et al., 2021; Paton et al., 2021), will not be included. However, understanding the signaling mechanisms underlying the adverse effects of KOR will be helpful for drug development of KOR agonists for all the indications.

## 2. Roles of G proteins and arrestins in kappa opioid receptor-mediated behaviors

### 2.1. Results from G-protein-coupled receptor kinase 3 and p38 mitogen-activated protein kinase knockout mice

Bruchas et al. (2006) reported that in cultured cells U50,488H increased phosphorylation of p38 mitogen-activated protein kinase (MAPK) via KOR-mediated mechanisms, which was shown to be dependent on GRK3-mediated KOR phosphorylation at S369 and β-arrestin recruitment. Bruchas et al. (2007) subsequently demonstrated that repeated swim stress caused activation of both KOR and p38 MAPK co-expressed in GABAergic neurons in the nucleus accumbens, cortex, and hippocampus and that activation of p38 MAPK was KOR dependent. The p38 inhibitor SB203580 blocked U50,488H-induced CPA and the KOR-dependent swim stress-induced immobility but did not affect KOR analgesia or non-selectively impact on associative learning. Mice lacking GRK3 did not show increased p38 phosphorylation after KOR activation, display swim stress-induced immobility, or develop CPA to U50,488. These results indicate that GRK3-dependent activation of p38 MAPK signaling by the KOR system mediates KOR agonist- or stress-induced aversion. Land et al. (2009) found that dopamine-deficient mice were still able to show significant CPA to U50,488. Micro-injection of the KOR antagonist norBNI into the dorsal raphe nucleus (DRN) blocked U50,488-induced CPA and stress-induced reinstatement of cocaine conditioned place preference (CPP). KOR-/- mice did not displayed CPA to U50,488H and expression by viral injection into the DRN of the wildtype KOR, but not the S369A KOR mutant, restored CPA to U50,488H. These results indicate that U50,488-induced CPA was encoded by a projection from the DRN to the nucleus accumbens (NAc). Ehrich et al. (2015) further reported that conditional deletion of KOR or p38alpha MAPK in dopaminergic neurons in the ventral tegmental area (VTA) blocked CPA to U50,488. In KOR-/- mice virus-mediated expression of the wildtype KOR, but not S369A KOR mutant, in the VTA dopamine neurons restored CPA to U50,488H. Although p38alpha MAPK inactivation blocked U50,488-CPA, it did not inhibit evoked dopamine release in the NAc. In contrast, KOR activation acutely inhibited the firing of VTA dopaminergic neuron, and repeated KOR activation attenuated this response. Conditional deletion of p38alpha MAPK blocked this adaptation and KOR-induced phosphorylation of Tyr12 of the GIRK subunit Kir3.1 in VTA dopaminergic neurons. Tyr12 phosphorylation of GIRK enhanced channel deactivation, thus reduced KOR responses. These results suggest that KOR-induced CPA requires regulation of VTA dopaminergic neuron excitability through a p38alpha MAPK effect on GIRK.

### 2.2. Results from β-arr2 knockout mice

White et al. (2015) examined effects of β-arr2 deletion on behavior effects induced by three KOR agonists: U69,593, salvinorin A (SalA) and RB-64. U69,593 is one of the prototypic KOR agonists. SalA is the first non-nitrogenous KOR agonist isolated from the mint plant *Salvia divinorum* and is highly selective for the KOR (Roth et al., 2002). RB-64 is a SalA analog (Yan et al., 2009). While U69,593 and SalA were reported to be unbiased agonists, RB-64 was a G protein-biased agonist (White et al., 2014).

**U69,593 and SalA**:β-arr2 deletion in mice reduced U69,593- or SalA-induced motor incoordination in the rotarod test, but did not affect U69,593- and SalA-produced analgesic effect in the hotplate test, CPA, or inhibition of novelty-induced hyperlocomotion. These researchers also showed that in β-arr2 knockout mice, U69,593 or SalA lowered the threshold of intracranial self-stimulation (ICSS) compared with the wildtype mice; however, at the doses that increased the ICSS threshold, U69,593 and SalA also reduced the maximal response rate. The latter response suggested hypolocomotion, thus confounding the data interpretation. Morgenweck et al. (2015) reported that there was no difference between wildtype and β-arr2 knockout mice in U50,488H-induced attenuation of chloroquine-induced scratching, suggesting that β-arr2 does not play a role in U50,488H-induced anti-scratch effects. These results indicate that β-arr2 is involved in KOR-induced motor incoordination in the rotarod test, but not analgesia, anti-itch, CPA, or hypolocomotion. The observation that β-arr2 is not involved in KOR-mediated CPA is different from those of Chavkin et al. (Bruchas et al., 2006, 2007; Brust, 2022; Land et al., 2009; Bruchas and Chavkin, 2010; Ehrich et al., 2015). Some caveats should be taken into consideration. β-arr2 deletion may result in compensatory changes. First, β-arr1 may fill in the roles normally played by β-arr2. Secondly, as β-arr2 is instrumental in signaling and regulation of many GPCRs, effects of constitutive β-arr2 deletion may be attributed to a combination of effects of β-arr2 deletion on the KOR and other GPCRs in the neuronal circuitry involved in the KOR-mediated behavior.

**RB-64**: RB-64 produced analgesic effects and CPA but did not impair rotarod performance or inhibit novelty-induced hyperlocomotion. Importantly, β-arr2 deletion did not affect any of RB-64 effects. RB-64 did not increase the ICSS threshold at doses that did not affect the maximal response rate. The observation that RB-64 caused CPA but did not change the ICSS threshold demonstrates the complexity of modeling KOR-mediated dysphoria (see section “7.1. Dysphoria” for more discussion).

### 2.3. Results from mice expressing phosphorylation-deficient kappa opioid receptor (K4A mice)

Chen et al. (2016) determined that U50,488H promoted phosphorylation of the mouse KOR at S356, T357, T363 and S369 in the C-terminal domain in cultured cells. A mouse line was generated that expressed a mutant KOR with all the four amino acid residues mutated to alanines (K4A) (Huang et al., 2022). K4A mutations abolished U50,488H-induced KOR phosphorylation at T363 and S369 in mouse brains as determined by western blotting with phospho-KOR specific antibodies that specifically recognized pT363 KOR and pS369 KOR (Chen et al., 2016, 2022b). U50,488H-promoted KOR phosphorylation at S356 and T357 occurred to much lower extents than at T363 and S369 and phosphorylation of T363 and S369 occurred before that of S356 and T357, thus we focused on T363 and S369 phosphorylation. Therefore, the K4A mouse line essentially expresses a G protein-biased KOR mutant. Compared with the wildtype mice, K4A mice exhibited similar U50,488H-induced anti-scratch activity and hypolocomotion in both males and females. In contrast, K4A mutations attenuated tolerance to the anti-scratch effect of U50,488H (5 mg/kg, s.c.) induced by repeated administrations of a high dose (80 mg/kg, i.p.) of U50,488H in male mice but not in female mice. In addition, K4A substitutions did not affect CPA induced by U50,488H (2 or 5 mg/kg) in male mice, but blunted U50,488H (2 mg/kg)-promoted CPA in female mice. Surprisingly, in female wildtype or K4A mice, 5 mg/kg U50,488H did not cause CPA. These results indicate that agonist-induced KOR phosphorylation has sex- and behavior-dependent effects. Agonist-promoted KOR phosphorylation is associated with tolerance in male mice only and with CPA in female mice only. In both males and females, KOR phosphorylation is unrelated to hypolocomotion or anti-scratch effect.

The finding that K4A mutations did not affect U50,488H-induced CPA in male mice is consistent with the study of White et al. (2015), who showed that β-arr2 deletion had no effects on CPA induced by the KOR agonists U69,593, salvinorin A and RB-64 in mice of mixed sexes (an equal number of male and female mice were age-matched). However, Bruchas et al. reported that, in male mice, KOR agonist-induced CPA was GRK3- and p38 MAPK-dependent, which was proposed to involve a β-arrestins-dependent signaling pathway (Bruchas et al., 2007, 2011; Ehrich et al., 2015).

In contrast, corroborative with the finding that in female mice, phosphorylation-deficient KOR (K4A) abolished U50,488H (2 mg/kg)-induced CPA, U50,488H-induced CPA in female mice was also proposed to be mediated by activation of β-arrestin and p38 MAPK similarly as in male mice by Chavkin et al. (Abraham et al., 2018).

### 2.4. Studies on agonists that have differential abilities to cause kappa opioid receptor phosphorylation: Lack of connection between kappa opioid receptor phosphorylation and conditioned place aversion

While results obtained from mutant mouse lines are informative, the possibility cannot be excluded that some unforeseen genetic or compensatory events may occur, which may confound interpretation of the data. Chen et al. (2022a) examined four KOR agonists for their abilities to promote KOR phosphorylation and internalization in mice and investigated whether there was a connection between phosphorylation or internalization and the side effects they produced, particularly CPA. U50,488H is a prototypic KOR agonist (Von Voigtlander et al., 1983). Methoxymethyl salvinorin B (MOM-SalB) is a long-acting analog of salvinorin A (Wang et al., 2008). Nalfurafine, a 4,5-epoxymorphinan analog first synthesized by Nagase et al. (Seki et al., 1999), is the first KOR agonist approved for clinical use in humans. At therapeutic doses, psychotomimetic effects and dysphoria were not reported as major adverse events. 42B, 3-dehydroxy analog of nalfurafine, was also first synthesized by Nagase et al. and found to be a moderately selective KOR agonist (Nagase and Fujii, 2013; Cao et al., 2020).

U50,488H, MOM-SalB, nalfurafine and 42B produced analgesic effects in the formalin test and inhibited compound 48/80-induced scratching in dose-dependent manner and their A_50_ values were calculated (Table 1). In the dose ranges producing analgesic and anti-scratch effects, U50,488H and MOM-SalB produced CPA independent of doses, whereas nalfurafine and 42B did not cause CPA, even at the doses that produced the maximal analgesic and anti-scratch effects.

**TABLE 1:** Summary of effects of the four KOR agonists on behaviors and KOR phosphorylation in male CD-1 mice and KOR internalization in male KOR-tdT mice.

	U50,488H	MOM-SalB	nalfurafine	42B
Analgesia in the formalin test (A_50_ value)	Yes (0.58 mg/kg)	Yes (17.0 μg/kg)	Yes (5.8 μg/kg)	Yes (2.08 mg/kg)
Inhibition of 48/80-induced scratch (A_50_ value)	Yes (2.07 mg/kg)	Yes (70.2 μg/kg)	Yes (8.0 μg/kg)	Yes (2.95 mg/kg)
CPA (doses used)	Yes (0.25–10 mg/kg)	Yes (10–300 μg/kg)	No (0.25–20 μg/kg)	No (1, 3, 5 mg/kg)
Motor incoordination (dose(s) used)	Yes (5 mg/kg)	Yes (200 μg/kg)	Slight effect (20 μg/kg)	Yes (3, 5 mg/kg)
Inhibition of novelty-induced hyper-locomotion (dose used)	Yes (5 mg/kg)	Yes (200 μg/kg)	No (20 μg/kg)	Yes (5 mg/kg)
KOR phosphorylation in the brain (dose used)	Yes (5 mg/kg)	Yes (200 μg/kg)	No (20 μg/kg)	Yes (5 mg/kg)
KOR internalization in the VTA (dose used)	Yes (5 mg/kg)	Yes (200 μg/kg)	No (20 μg/kg)	Yes (5 mg/kg)

Results of CPA, motor incoordination, hypolocomotion, KOR phosphorylation, and KOR internalization were obtained at doses (in parentheses) that produce maximal analgesic effect in the formalin test and maximal inhibitory effects on compound 48/80-induced scratch behavior [Reproduced from Chen et al. (2022a)].

For the rotarod test, inhibition of novelty-induced hyperlocomotion and KOR phosphorylation and internalization, each drug was administered in the dose range producing effective analgesic and anti-scratch effects, including a dose that reached the maximal effects in the dose-response relationship.

U50,488H, MOM-SalB and 42B impaired rotarod performance and inhibited novelty-induced hyperlocomotion, but nalfurafine did not inhibit novelty-induced hyperlocomotion and caused only slight impairment in rotarod performance (Table 1).

For agonist-promoted KOR phosphorylation studies, mice were injected with one drug at an appropriate dose or vehicle. Thirty minutes later, mice were sacrificed, and brains were removed. Four brains were pooled as one sample because of the low KOR level, KOR was enriched with immunoprecipitation and phosphorylated KOR was detected with immunoblotting with antibodies against phospho-T363 KOR and phospho-S369 KOR. U50,488H, MOM-SalB and 42B promoted robust phosphorylation at T363 and S369, but nalfurafine did not cause phosphorylation.

For agonist-induced KOR internalization, a mutant mouse line expressing the KOR conjugated with the fluorescent protein tdTomato (KtdT) was used. Mice were injected with a drug at the same dose as in the phosphorylation studies and 30 min later perfused for immunohistochemistry (IHC) with antibodies against tdTomato. U50,488H, MOM-SalB and 42B, but not nalfurafine, promoted KOR internalization in the VTA.

The results that U50,488H and MOM-SalB, but not nalfurafine or 42B, caused CPA and that U50,488H, MOM-SalB, and 42B, but not nalfurafine, cause KOR phosphorylation suggest that there is a lack of a one-to-one connection between KOR phosphorylation and CPA. U50,488H, MOM-SalB and 42B, but not nalfurafine, promoted KOR phosphorylation and KOR internalization, suggesting that KOR phosphorylation and KOR internalization are related, consistent with the traditional concept that agonist-promoted KOR phosphorylation precedes KOR internalization. The observations that 42B causes motor incoordination and inhibition of novelty-induced hyperlocomotion but not CPA, indicates that neuronal circuits and signaling pathways associated with these behaviors are different. From the perspective of drug discovery, a KOR full agonist that does not promote KOR phosphorylation in the mouse brain or KOR internalization in the VTA in the dose range causing full analgesic effects (such as nalfurafine) may cause fewer side effects or lower levels of side effects. Thus, lack of agonist-promoted KOR phosphorylation or internalization may be used as a screening method. On the other hand, a KOR full agonist that promotes KOR phosphorylation or internalization (such as 42B) may not cause CPA but still produce hypolocomotion and motor incoordination.

## 3. Studies on nalfurafine, a clinically used and centrally acting kappa opioid receptor agonist

### 3.1. *In vitro* characterization

Nalfurafine was first synthesized by Nagase et al. (1998). *In vitro* it was found to be a highly potent KOR full agonist and MOR partial agonist with a moderate selectivity for the KOR over MOR and low affinity for the delta opioid and nociceptin/orphanin FQ receptors (DOR and NOR, respectively) (Seki et al., 1999; Wang et al., 2005; Nagase and Fujii, 2013; Che et al., 2018; Cao et al., 2020; reviewed in Zhou et al., 2022). It did not show significant affinity for 45 biochemical targets screened, other than opioid receptors (Nakao and Mochizuki, 2009; reviewed in Zhou et al., 2022).

### 3.2. *In vivo* characterization in rodents

In mice, nalfurafine produces analgesic effect in the formalin test, acetic acid-induced abdominal constriction test, 51°C hot plate, focused heat tail flick, mechanical tail pressure and tail pinch tests (Endoh et al., 1999; Liu et al., 2019). Among these tests, nalfurafine is much less active in the 51°C hot plate and focused heat tail flick. In addition, nalfurafine inhibited scratching behaviors induced by compound 48/80, chloroquine, histamine, substance P, the KOR antagonist 5’-GNTI, the protease-activating receptor 2 agonist SLIGRL, and serotonin (Togashi et al., 2002; Inan and Cowan, 2004; Wang et al., 2005; Kardon et al., 2014; Snyder et al., 2018; Liu et al., 2019; reviewed in Zhou et al., 2022). In the dose ranges effective for analgesic and anti-itch effects, nalfurafine did not cause CPA or inhibition of novelty-induced hyperlocomotion, but it did cause slight but significant impairment in motor coordination (Tsuji et al., 2001; Mori et al., 2002; Liu et al., 2019; Zhou and Kreek, 2019; reviewed in Zhou et al., 2022). In addition, nalfurafine did not promote KOR phosphorylation in the mouse brain or KOR internalization in the mouse VTA (Chen et al., 2022a). These data are by and large consistent with the observations in humans. It should be noted, however, at higher doses, nalfurafine did cause CPA and hypolocomotion in mice (Endoh et al., 1999; Mori et al., 2002; Togashi et al., 2002; Kaski et al., 2019).

### 3.3. *In vitro* agonist bias

Whether nalfurafine is a biased agonist *in vitro* has been examined by several laboratories and the results are summarized in Table 2. It has been demonstrated to be G protein-biased, unbiased, and β-arrestin-biased. The variations are likely due to differences in experimental conditions used, including the cell line, assays for G protein and β-arrestin signaling and methods for calculation of agonist bias.

**TABLE 2:** Determination of *in vitro* agonist biases of nalfurafine [Reproduced from (Zhou et al., 2022)].

References	G protein signaling; cells used	β-arrestin signaling; cells used	Agonist bias (Reference compound)
Schattauer et al. (2017)	ERK1/2 phosphorylation; HEK293 cells stably expressing rat KOR-GFP	P38 MAPK phosphorylation; HEK293 cells stably expressing rat KOR-GFP	G protein-biased, 7.2 (U50,488H)
Schattauer et al. (2017)	ERK1/2 phosphorylation; HEK293 cells stably expressing FLAG-human KOR	P38 MAPK phosphorylation; HEK293 cells stably expressing FLAG-human KOR	G protein-biased, 300 (U50,488H)
Liu et al. (2019)	[^35^S]GTPγS binding; neuro2A cells stably expressing mouse KOR	γ-galactosidase fragment complementation; HEK cells stably expressing mouse KOR-donor and β-arrestin1 or 2-acceptor	Unbiased (U50,488H)
Dunn et al. (2019)	[^35^S]GTPγS binding; human KOR-expressing U2OS cells (PathHunter, DiscoveRx)	γ-galactosidase fragment complementation; human KOR-expressing U2OS cells (PathHunter, DiscoveRx)	β-arrestin-biased (U69,593)
Kaski et al. (2019)	GloSensor (cAMP); HEK293T cells transiently transfected with 3 × HA-hKOR and pGloSensor-22F cAMP biosensor	Tango luciferase-based assay; HTLA cells transiently transfected with human KOR-V2-TEV-tTA	G protein-biased, 7.7 (U50,488)
Cao et al. (2020)	GloSensor (cAMP); HEK293-T cells stably expressing human KOR were transfected with GloSensor for cAMP	Tango β-arrestin2 recruitment; HEK293 cells stably expressing a tTA-dependent luciferase reporter and a β-arrestin2-TEV fusion gene were transfected with the human KOR	G protein-biased, 2.85 (U50,488H)

### 3.4. Distinct phosphoproteomic changes in mouse brains by nalfurafine vs. U50,488H

Taking advantage of the differences between U50,488H and nalfurafine in inducing CPA, hypolocomotion and motor incoordination, Liu et al. (2019) examined the effects of the two drugs on downstream signaling in CD-1 mouse brains by a large-scale unbiased phosphoproteomic approach. Male CD-1 mice were treated with saline, U50,488H (5 mg/kg, s.c.) or nalfurafine (30 μg/kg, s.c.) for 30 min, a time point when their pharmacological effects were fully manifested. Brains were removed and four brain regions (cerebral cortex, striatum, hippocampus, and spinal cord) were dissected and frozen. Each sample was processed for phosphoproteomics. Principal component analysis showed that there were brain region-specific changes in phosphoproteome and that U50,488H and nalfurafine produced different phosphoproteomic changes in the cortex and striatum, but not in the hippocampus or spinal cord. Data analyzed using the Kyoto Encyclopedia of Genes and Genomes (KEGG) pathway database showed that many signaling pathways were differentially regulated by the two agonists, including the Notch signaling pathway, Wnt signaling pathway, regulation of actin cytoskeleton and mTOR pathway. It is noteworthy that the data analyses were done at the signal pathway level, and not all phosphorylation states of the signaling molecules could be captured at a single time point. The mTOR pathway in the cortex and striatum was activated by U50,488H but not by nalfurafine. Inhibition of the mTOR pathway with rapamycin attenuated U50,488H- and MOM-SalB-induced CPA, but did not affect U50,488H-produced analgesia, anti-scratch effect, hypolocomotion or motor incoordination. These results indicate that the mTOR pathway is involved in the KOR-mediated CPA, but not analgesia, anti-scratch effect, hypolocomotion or motor incoordination. Thus, among the three undesirable effects of U50,488H, the mTOR pathway is involved in CPA, but not hypolocomotion or motor incoordination.

However, significant modulation of the p38 MAPK pathway was not detected in male mouse brains at 30 min after U50,488H treatment by the large-scale phosphoproteomic approaches (Liu et al., 2019, 2020). No change was found at 5 min either (see below).

Liu et al. (2019) attempted to determine if in the mouse striatum, U50,488H and nalfurafine caused differential phosphorylation of p70S6K, one of the kinases in the mTOR pathway, with immunoblotting using antibodies against phospho-p70S6K at T389 and p70S6K. Immunoblotting results showed that phospho-p70S6K had an Mr of 85 kDa, whereas the p70S6K had an Mr of 70 kDa. The discrepancy in the relative molecular weights of the phosphorylated and unphosphorylated forms of p70S6K made it impossible to interpret the data. These results precluded the use of western blot of phospho-p70S6K as a screening method for KOR agonists that do not cause CPA. We have also examined whether *in vitro* U50,488H and nalfurafine differentially cause phosphorylation of p70S6K. We found that both agonists cause phosphorylation of p70S6K at T389 to similar extents and curiously, phosphorylated p70S6K and unphosphorylated p70S6K in neuro2A cells stably transfected with the mouse KOR had an Mr of 70 kDa, different from those in the mouse striatum.

## 4. Large-scale unbiased phosphoproteomic studies: mTOR pathway and CB1 cannabinoid receptors are involved in kappa opioid receptor-mediated conditioned place aversion

Protein phosphorylation and de-phosphorylation play critical roles in regulating activities of proteins (such as enzyme activities), association with interacting proteins and co-factors, and are important in signaling cascades leading to eventual cellular responses. Liu et al. (2018, 2019, 2020) performed a series of high-throughput unbiased phosphoproteomic studies to elucidate signaling pathways that may be involved in KOR-mediated side effects, particularly CPA.

### 4.1. Distinct phosphoproteomic changes induced by U50,488H and nalfurafine

See section “3.4. Distinct phosphoproteomic changes in mouse brains by nalfurafine vs.U50,488H.”

#### 4.2. Differential regulation of phosphoproteomes in mouse brains by CPA-inducing and non-CPA-inducing KOR agonists

Liu et al. (2018) performed high-throughput large-scale phosphoproteomic studies on five brain regions (cortex, hippocampus, striatum, cerebellum, and medulla oblongata) of mice treated with the KOR agonists U50,488H, HS665, HS666, RB-64 and 6’-GNTI. Both HS665 and HS666, which are diphenethylamine derivatives, were determined to be G protein-biased KOR full agonist and partial agonist, respectively (Spetea et al., 2017). 6’-Guanidinonaltrindole (6’-GNTI) was found to be a potent KOR partial agonist at activating the G proteins. 6’-GNTI did not recruit arrestin and blocked arrestin recruitment and KOR internalization induced by other non-biased agonists (Rives et al., 2012). The five brain regions express different levels of the KOR. Among the agonists, U50,488H, HS665, and RB-64 induced KOR-mediated CPA, whereas HS666 and 6’-GNTI did not. Following treatment with KOR agonists, Liu et al. (2018) identified >50,000 phosphorylation sites, and by use of dynorphin- and KOR-knockout mice as the controls, they determined the phosphorylation sites specifically related to KOR activation. Each brain region of control mice showed distinct phosphoproteomes and treatment with KOR agonists yielded distinct phosphoproteomic changes in each region. Five-min and 30-min treatments with the same agonist produced different changes. Further analysis revealed that KOR-mediated phosphoproteomic changes occur to specific neurotransmitter-expressing neuronal circuits, especially dopamine-, glutamate-, and γ-aminobutyric acid (GABA)- and cholin-ergic signaling, indicating that through synapse and circuitry connections KOR activation impacts on signaling of these neurons. Many of the signaling molecules are phosphorylated or dephosphorylated, indicating that both phosphatases and kinases are involved in the synaptic signaling transduction pathways utilized by KOR agonists. Some examples of signaling molecules and phosphorylation sites affected by U50,488H are ERK1/2 (T203/Y203), DARPP-32 (S97), Src (S17, S74), Raf1 (S259), CB1R (S317), NMDAR2a (S1459), Gng12 (S7). Pathway analyses of phosphoproteomic changes revealed that among the pathways activated by CPA-producing KOR agonists but not by non-CPA-producing agonists is the mTOR pathway. Pretreatment with the mTOR inhibitor temsirolimus abolished U50,488H-induced CPA in mice, similar to the finding of Liu et al. (2019) (see section “3.4. Distinct phosphoproteomic changes in mouse brains by nalfurafine vs. U50,488H”). In contrast, mTOR inhibition had no effects on U50,488H-induced anticonvulsant and antinociceptive effects, suggesting that mTOR pathways are involved in the side effects but not therapeutic actions of KOR opioids. Such phosphoproteomics studies reveal the complex phosphorylation and dephosphorylation signaling cascades that occur following KOR activation in the brain and will pave ways for more comprehensive understanding of signaling involved in KOR-mediated behaviors.

### 4.3. Protein kinases C are involved in kappa opioid receptor-mediated adverse effects

Liu et al. (2020) reported that pretreatment of male CD-1 mice with chelerythrine, a non-selective protein kinase C (PKC) inhibitor that inhibits classical PKCs (α, β and γ) and novel PKCs (δ, ϵ, η and θ), attenuated CPA, sedation, and motor incoordination induced by the KOR agonist U50,488H but did not affect its antinociceptive and antipruritic effects. PKC inhibition with chelerythrine attenuated U50,488H-induced KOR phosphorylation at Ser369, but did not affect phosphorylation at Thr363, in the male mouse brain. A large-scale unbiased phosphoproteomic study on mouse brains was performed. Pretreatment with chelerythrine affected U50,488H signaling differently at 5 and 30 min after U50,488H administration. At 5 min, U50,488H modulated GRKs and Wnt pathways in a PKC-dependent manner. At 30 min U50,488H regulated stress signaling, cytoskeleton, mTOR signaling and receptor phosphorylation in a PKC-dependent fashion. Among the receptors of which phosphorylation levels were changed was the CB1 cannabinoid receptor. The CB1 neutral antagonist AM4113 attenuated U50,488H-induced CPA, indicating that CB1 receptor is involved in KOR-mediated CPA (Liu et al., 2020). Thus, PKC appears to play an important role in KOR-mediated adverse effects and further phosphoproteomic studies may delineate in more details the signaling pathways involved in each effect.

## 5. Novel G protein-biased kappa opioid receptor agonists

Novel G protein-biased KOR agonists have been identified by *in vitro* assays and those that have effective antinociceptive or anti-scratch effects are discussed here, including triazole 1.1 (Zhou et al., 2013), isoquinolinone 2.1 (Zhou et al., 2013), RB-64 (White et al., 2014, 2015), HS665 and HS666 (Spetea et al., 2017), 16-Bromo SalA (Paton et al., 2020) and LOR-17 (Bedini et al., 2020). Other novel G protein-biased KOR agonists that did not produce effective analgesia or were not studied for antinociceptive effects are not included, such as mesyl Sal B (Kivell et al., 2018), ethoxymethyl ether Sal B and β-tetrahydropyran Sal B (Ewald et al., 2017). Readers are referred to a recent comprehensive review on G protein-biased KOR agonists by French and Van Rijn (2022).

### 5.1. *In vitro* characterization

The *in vitro* assays used, and bias factors calculated from the *in vitro* data of these compounds are summarized in Table 3.

**TABLE 3:** *In vitro* agonist biases of novel G protein-biased KOR agonists which were investigated *in vivo* for analgesic or anti-scratch effects.

Compound (Reference)	G protein signaling; cells used	β-arrestin signaling; cells used	Agonist bias (Reference compound)
Triazole 1.1 (Zhou et al., 2013)	1. Membrane [^35^S]GTPγS binding (mG); CHO-hKOR cells 2. Whole Cell [^35^S]GTPγS binding (wcG); CHO-hKOR cells 3. Cellular Impedance; CHO-hKOR cells	β-Arrestin2 recruitment; DiscoverX PathHunter U2OS EFC cell line expressing β-arrestin2 and hKOR β-Arrestin2 Imaging; U2OS cell line stably expressing hKOR and β-arrestin2-eGFP	(U69,593) mG/β-arr2 EFC, 61.2 mG/β-arr2 imaging, 20.0 wcG/β-arr2 EFC, 8.6 wcG/β-arr2 imaging, 2.8 imped/β-arr2 EFC, 6.9 imped/β-arr2 imaging, 2.3
Isoquinolinone 2.1 (Zhou et al., 2013)			(U69,593) mG/β-arr2 EFC, 31.4 mG/β-arr2 imaging, 7.2 wcG/β-arr2 EFC, 17.3 wcG/β-arr2 imaging, 4.0 imped/β-arr2 EFC, 66.7 imped/β-arr2 imaging, 3.0
Triazole 1.1 (Brust et al., 2016)	Membrane [^35^S]GTPγS binding (mG); CHO-hKOR cells	β-Arrestin2 recruitment (DiscoveRx PathHunter) U2OS EFC cell line expressing β-arrestin2 and hKOR	(U50,488H) G protein-biased, 28
RB-64 (White et al., 2014)	cAMP, inhibition of isoproterenol-stimulated adenylate cyclase (GloSensor); HEK293T cells transiently transfected with 3 × HA-hKOR and pGloSensor-22F cAMP biosensor	β-arrestin2 recruitment (Tango luciferase-based assay); HTLA cells transiently transfected with human KOR-V2-TEV-tTA	G protein-biased, 35 (Salvinorin A)
HS665, HS666 (Spetea et al., 2017)	[^35^S]GTPγS binding; CHO cells stably expressing the hKOR	β-arrestin2 recruitment assay (DiscoveRx PathHunter); U2OS cells stably co-expressing the hKOR and the enzyme acceptor-taggedβ-arrestin2 fusion protein	HS665, G protein-biased, 389 HS666, G protein-biased, 62 (U69,593)
16-Bromo SalA (Paton et al., 2020)	Inhibition of forskolin-stimulated adenylate cyclase (DiscoveRx HitHunter)	β-arrestin2 recruitment assay (DiscoveRx PathHunter)	G protein-biased, 7.7 (U50,488H)
LOR17 (c[Phe-Gly-(b-Ala)-DTrp]) (Bedini et al., 2020)	Inhibition of forskolin-stimulated adenylate cyclase (cAMP ELISA assay), HEK-293 cells expressing the human KOR, U87-MG cells and normal human astrocytes	β-arrestin2 recruitment assay (DiscoveRx PathHunter) with U2OS cells co-expressing the human KOR and the enzyme acceptor tagged β-arrestin 2 fusion protein	G protein-biased, 853 (U50,488H)
LOR17 (c[Phe-Gly-(b-Ala)-DTrp]) (Bedini et al., 2020)	Early-phase ERK1/2 phosphorylation	p38MAPK phosphorylation; p38MAPK-mediated cell proliferation in astrocytes	No bias calculation

### 5.2. Behavior effects of novel G protein-biased agonists

The results on novel G protein-biased KOR agonists that have effective antinociceptive or anti-scratch effects are described below and summarized in Table 4.

**TABLE 4:** *In vivo* pharmacological characterization of some G protein-biased KOR agonists in rodents.

Compound (Reference)	Analgesia	Antipruritic effect	CPA	ICSS	Hypolo-comotion	rotarod performance	Additional effect
RB-64 (dose) (White et al., 2015) mice	Yes (55°C hot plate) (3 & 10 mg/kg)		Yes (1 & 3 mg/kg)	No effect (1 mg/kg)	No effect (3 mg/kg)	No effect (3 & 10 mg/kg)	
Triazole 1.1 (dose) (Brust et al., 2016), mice except ICSS	Yes (49°C warm water tail immersion) (15, 30 mg/kg)	Yes (against chloroquine) (1, 3 mg/kg)		No effect in rats (4, 8, 16, 24 mg/kg)	No (3, 15 & 30 mg/kg)		No effect on dopamine release in the N accumbens
Isoquinolinone 2.1 (dose) (Morgenweck et al., 2015), mice		Yes (against norBNI or chloroquine) (1, 3 mg/kg)			No effect (3 mg/kg)		
HS 665 (dose) (Spetea et al., 2017)	Yes, 55°C warm-water tail withdrawal, ED_50_ 3.74 nmole, icv		Yes 30 nmole, icv		No effect 10 nmole, icv		
HS 666 icv (dose) (Spetea et al., 2017)	Yes, 55°C warm-water tail withdrawal, ED_50_ 6.02 nmole, icv		No 150 nmole, icv		No effect, 30 nmole, icv		
16-Bromo SalA (Paton et al., 2020), mice	Yes, 50°C warm-water tail withdrawal, ED_50_, 2.06 mg/kg; not effective in 2% formaldehyde Induced inflammatory pain at 1, 2 mg/kg		No, 0.3 mg/kg; no conditioned taste aversion, 0.3 mg/kg			Impaired performance at 2 mg/kg, but not at 1 mg/kg	No axiogenic effect in the elevated plus maze test (1, 2 mg/kg)
LOR17 (dose) (Bedini et al., 2020)	Yes, 52°C warm water tail immersion, ED_50_ 10.07 mg/kg, sc; acetic acid abdominal writhing, ED_50_ 5.74 mg/kg. sc)				No (10 mg/kg, sc)	No (10 mg/kg, sc)	Attenuated oxaliplatin-induced cold hypersensitivity (10 and 20 mg/kg, sc); No effect in the forced swim test (10 mg/kg, s.c.)

White et al. (2015) showed that in mice, RB-64 produced analgesia in the 55°C hot plate assay and CPA, but no inhibition of novelty-induced hyperlocomotion or anhedonia in the ICSS test. β-arr2 deletion has no effect on RB-64-induced behavior responses.

Brust et al. (2016) demonstrated that triazole 1.1 caused analgesia in the 49°C warm water tail immersion assay and antipruritic effect against chloroquine phosphate-induced scratching. In contrast to typical KOR agonists, it did not induce hypolocomotion or reductions in dopamine release in the nucleus accumbens in mice, nor did it cause dysphoria in the ICSS test in rats. Zamarripa et al. (2021) showed that in male rats triazole 1.1 reduced oxycodone self-administration and increased antinociceptive effect of oxycodone in a hot plate test. Huskinson et al. (2022) observed that in the rhesus monkey, triazole 1.1, nalfurafine and U50,488H all reduced oxycodone-induced scratching. However, U50,488H and nalfurafine, but not triazole 1.1, produced sedative-like and motor-impairing effects.

Isoquinolinone 2.1 was as effective as U50,488H in suppressing the itch response induced by the KOR antagonist NorBNI or chloroquine phosphate in C57BL/6J mice (Morgenweck et al., 2015).

Spetea et al. (2017) demonstrated that HS665 and HS666 (i.c.v.) exhibited KOR-mediated antinociceptive effect in the 55°C warm-water tail withdrawal assay with similar potencies as U50,488. At doses that produced the maximal antinociception, HS665 and HS666 (i.c.v.) did not alter locomotor activities; however, HS665 produced CPA, but HS666 did not.

LOR17 (i.p.) displayed potent antinociception in the hot water (52 ± 0.5°C) tail immersion test and in the acetic acid abdominal writhing test like U50,488H. LOR17 attenuated oxaliplatin-induced cold hypersensitivity better than U50,488, and it was effective after single or repeated s.c. administration. LOR17 administered at a dose that fully alleviated oxaliplatin-induced cold hypersensitivity did not alter motor coordination in the rotarod test or locomotor and exploratory activities in the hole-board test. Nor did it induce pro-depressant-like behavior in the forced swim test.

In summary, KOR agonists determined to have G protein signaling biases were shown to have analgesic and antipruritic effects and at the effective dose ranges, they did not cause hypolocomotion. However, their other pharmacological effects vary. RB-64 and HS665 produced CPA, whereas HS666 did not and LOR17 and isoquinolinone 2.1 were not tested. Triazole 1.1 and RB-64 did not affect stimulating threshold in the ICSS test and triazole 1.1 did not reduce dopamine release in the nucleus accumbens. LOR17 and RB-64 did not cause motor incoordination in the rotarod test. Thus, generally speaking, G protein-biased KOR agonists appear to have better pharmacological profiles than prototypical KOR agonists such as U50,488H. However, some of them still produce side effects. These may be due to differences in agonist bias determination and pharmacokinetic issues, including metabolism to compounds with different pharmacological profiles from the parent compounds. To the best of our knowledge, none of the G protein-biased KOR agonists have proceeded to clinical trials yet.

## 6. Different mechanisms are involved in kappa opioid receptor-mediated conditioned place aversion, hypolocomotion, and motor incoordination

In this section, only studies that examined at least two of the three behavior end points are discussed (Tables 5, 6).

**TABLE 5:** Effects of inhibition of signaling molecules or genetic manipulations on KOR agonist-induced CPA, hypolocomotion and motor incoordination.

Inhibition of signaling or genetic manipulation	CPA	Inhibition of novelty- induced hyperlocomotion	Motor incoordination in the rotarod test
PKC inhibition with chelerythrine (Liu et al., 2020)	Reduced CPA	Reduced hypolocomotion	Reduced motor incoordination
mTOR inhibition (Liu et al., 2018, 2019)	Inhibited CPA	No effect	No effect
β-arrestin2-/- mice (White et al., 2015)	Same as the WT	Same as the WT	Lower than the WT
K4A mice (Huang et al., 2022)	Males, Same as the WT; Females, lower than the WT	Males and females, same as the WT	ND

ND, not determined.

**TABLE 6:** Effects of KOR agonists on CPA, hypolocomotion and motor incoordination.

KOR agonists	CPA	Inhibition of novelty-induced hyperlocomotion	Motor incoordination in the rotarod test
RB-64 (White et al., 2015)	Yes	No	No
Nalfurafine (20 μg/kg) (Liu et al., 2019)	No	No	Small but significant effect
3-deoxyl nalfurafine (42B) (Chen et al., 2022a)	No	Yes	Yes
16-Bromo Sal A (Paton et al., 2020)	No	No	ND
HS666 (Spetea et al., 2017)	No	No	ND
HS665 (Spetea et al., 2017)	Yes	No	ND
LOR17 (Bedini et al., 2020)	ND	No (in the hole-board test)	No

ND, not determined.

Inhibition of PKC or the mTOR pathway, deletion of β-arrestin2, or mutation of KOR phosphorylation sites have distinct effects on the three behaviors (Table 5). None of these treatments or genetic manipulations affected antinociceptive or anti-scratch effects produced by KOR agonists. Inhibition PKC by chelerythrine in male mice attenuated U50,488H-induced CPA, inhibition of novelty-induced hyperlocomotion, and motor incoordination (Liu et al., 2020), demonstrating that PKC is involved in all the three behaviors. Inhibition of the mTOR pathway by rapamycin in male mice reduced U50,488H-promoted CPA, but had no effect on inhibition of novelty-induced hyperlocomotion, or motor incoordination, showing that the mTOR pathway is involved in CPA only (Liu et al., 2018, 2019). β-arrestin2 deletion in a mixed population of male and female mice reduced U69,593 or salvinorin A-produced motor incoordination in the rotarod test, without affecting CPA or inhibition of novelty-induced hyperlocomotion, indicating that β-arrestin2 plays an important role in motor incoordination, but not the other two effects (White et al., 2015). In K4A male mice, KOR phosphorylation deficiency did not affect CPA or inhibition of novelty-induced hyperlocomotion; however, in K4A female mice, KOR phosphorylation deficiency attenuated U50,488H-induced CPA without impacting on inhibition of novelty-induced hyperlocomotion, demonstrating that agonist-promoted KOR phosphorylation is involved in CPA in females only (Huang et al., 2022). These results show that different signaling mechanisms are involved in KOR-mediated CPA, inhibition of novelty-induced hyperlocomotion, and motor incoordination.

All the KOR agonists examined have antinociceptive effects; however, KOR agonists have different abilities to produce CPA, inhibition of novelty-induced hyperlocomotion and motor incoordination (Table 6). U50,488H, MOM-SalB, U69,593 and salvinorin A produced all the three effects (White et al., 2015; Liu et al., 2019). 16-Bromo Sal B and HS666 did not induce CPA or inhibit novelty-induced hyperlocomotion (Spetea et al., 2017; Paton et al., 2020). HS665 elicited CPA but not hypolocomotion. RB-64 produced CPA, but not hypolocomotion or motor incoordination; in contrast, 42B caused profound hypolocomotion and motor incoordination but not CPA. Nalfurafine produced small but significant motor incoordination but not CPA or hypolocomotion. LOR17 did not cause hypolocomotion or motor incoordination (Bedini et al., 2020). These results further suggest that these three adverse behavior effects are mediated by different mechanisms.

## 7. Considerations on behavioral tests

### 7.1. Dysphoria

Dysphoria is one of the most important adverse effects of typical KOR agonists. CPA has been widely used as an animal correlate of dysphoria. However, dysphoria is an acute effect, whereas CPA requires at least three conditioning sessions pairing the compound of interest with the environmental cues and, thus, learning and memory are part of the conditioning process. CPA is useful for examining if a compound causes dysphoria/aversion, but it may not be a definitive assay for investigation of the signaling mechanisms underlying aversion itself, unless the signaling events are also proven to not be the basis of aversion-associated learning and memory.

ICSS has been used to determine if a KOR agonist causes dysphoria or anhedonia in rats or mice (Todtenkopf et al., 2004; Carlezon et al., 2006; Carlezon and Chartoff, 2007; Beguin et al., 2008; White et al., 2015; Brust et al., 2016; Liu et al., 2019; Huang et al., 2020). The stimulating electrode is placed in the VTA or the medial forebrain bundle and the animals undergo training to self-stimulate until stable stimulation threshold and rate are attained. Psychostimulants have been shown to lower the threshold for self-stimulation, whereas compounds causing dysphoria or anhedonia increase the threshold. White et al. (2015) reported that in mice, U69,593 and salvinorin A enhanced the ICSS threshold, but RB-64 had no effect. However, at most doses and time points (except for one salvinorin A dose at only one time point) in which the ICSS threshold was increased, the maximal response rate was decreased, indicating hypolocomotion. Brust et al. (2016) showed that in rats U50,488 increased the ICSS threshold, but Triazole 1.1 did not. A similar issue that at the U50,488H doses that ICSS threshold was increased, the maximal response rate was decreased, which confounded interpretation of the data. Liu et al. (2019) reported that U50,488H (2.5 mg/kg) increased the ICSS threshold and showed a trend of decreasing maximal wheel-spinning rates but did not reach statistical significance. Later, with more animals in each treatment group [*N* = 18 (Huang et al., 2020) vs 12 (Liu et al., 2019)], U50,488H at 2 mg/kg (s.c.), which produced robust CPA, did not affect the threshold, but significantly reduced the maximal response rate. It should be noted that the increase in ICSS thresholds caused by KOR agonists (e.g., U69,593 or U50,488H) may result from sedation/motor incapacitation induced by the agonists. Thus, whether ICSS can be used consistently and reliably to study dysphoria induced by KOR agonists is not without controversy.

### 7.2. Hallucination

Kappa opioid receptor activation causes depersonalization, perceptual distortions, and hallucinations (Pfeiffer et al., 1986). A prominent effect of ingesting leaves of the mint family plant *Salvia divinorum* in humans is hallucination and the active compound salvinorin A is a potent and selective KOR agonist (Roth et al., 2002). However, animal models of KOR-mediated hallucination have not been firmly established.

Prepulse inhibition (PPI) is a phenomenon in which a small stimulus prior to a larger reflex-inducing stimulus reduces the response to the latter stimulus and usually a startle reflex is used. The stimulation most often used is acoustic; however, light or tactile stimuli are also employed. PPI is used to measure sensorimotor gating and preattentional functions and reduced PPI is used as an animal model for cognitive deficits or hallucination (Geyer, 2006). Deficits in PPI are found in patients suffering from schizophrenia, mania or other psychiatric diseases (Swerdlow et al., 1999). Whether KOR activation reduces PPI has been examined, but the results are equivocal. Bortolato et al. (2005) reported that in rats, U50,488 (1–5 mg/kg, sc) dose-dependently reduced PPI of acoustic startle response, which was abolished by pretreatment with norBNI or the atypical antipsychotic drug clozapine but not by the typical antipsychotic drug haloperidol. In contrast, Tejeda et al. (2010) demonstrated that in rats, U50,488H (2.5 or 5.0 mg/kg, s.c.), U69,593 (0.16, 0.32 mg/kg, s.c.) or salvinorin A (1.0, 2.0 mg/kg, i.p.) did not affect PPI of acoustic startle reflex and that norBNI did not affect corticotropin releasing factor- or stress-induced deficits in PPI. Tejeda et al. (2010) speculated that the differences between the two studies may be due to different sources of Sprague-Dawley rats (European vs. American), since all the other experimental parameters were similar. Yan et al. (2009) observed that in mice, salvinorin A (2.0 mg/kg) or RB-64 (0.1 mg/kg) caused inhibition of PPI, which was reversed by norBNI; however, at lower doses neither compound produced consistent results.

Head twitch has been used as an animal correlate of hallucination in humans for compounds acting on the 5HT_2*A*_ receptor, such as lysergic acid diethylamide (LSD) and psilocybin (Halberstadt and Geyer, 2011). However, KOR agonists do not cause head twitch (Bryan Roth, personal communication).

### 7.3. Analgesic effects

Kappa opioid receptor agonists produce antinociceptive effects in animals. Several antinociception tests have been mentioned above, including the formalin test, acetic acid writhing test, hot plate test and warm water tail immersion test. Evidence in the literature reveals that KOR agonists are not efficacious in antinociceptive tests using heat as the stimulus and are effective only in certain tests, such as the paw pressure tests, abdominal constriction tests and formalin test.

Tyers (1980) examined MOR-preferring and KOR-preferring agonists in different antinociceptive tests. In the tests employing heat as the nociceptive stimuli (55°C hot plate test in mice and 55°C tail immersion test in rats), MOR agonists such as morphine, pethidine and D-propoxyphene exhibited steep and parallel dose-response curves and achieved the maximal responses without motor incapacitation. The potency ratios of these drugs in the heat tests were comparable with those for analgesia in humans. In contrast, KOR-preferring agonists, such as ethylketocyclazocine, ketocyclazocine, nalorphine and Mr1353, showed low or no efficacy in the heat tests and were only active at doses that caused sedation and motor impairment. In the acetylcholine-induced abdominal writhing test in mice and the paw pressure test in rats, both MOR agonists and KOR agonists produced steep and parallel dose response curves and achieved similar maximal responses.

After examining many opioid compounds in five antinociception tests, Hayes et al. (1987) concluded that the potencies of KOR agonists were in the order of the mouse acetylcholine abdominal constriction test = guinea-pig paw pressure test > rat paw pressure test > mouse light beam tail flick test > > mouse 55°C hot plate test. In addition, high efficacy MOR agonists were active in all the tests, whereas KOR full agonists were active in the mouse tail flick test but may not be truly antinociceptive in the hot plate test. These findings are consistent with the notion that MOR full agonists are more effective than KOR full agonists for treatment of acute pain. KOR partial agonists such as nalorphine were active only in the mouse abdominal constriction test and the guinea pig paw pressure test, but they were inactive in the mouse hot plate and paw pressure tests and required high doses to show activity in the mouse tail flick test. In the mouse hot plate test, most KOR full agonists were active only at doses ≥ their sedative ED_50_ in the rotarod test, while most KOR preferring partial agonists were inactive. Therefore, the warm water tail flick test and hot plate test may not be the most sensitive assays to detect analgesic effects of KOR agonists.

Endoh et al. (1999) demonstrated that nalfurafine (TRK-820) given s.c. showed antinociceptive activities in several tests in male ddY mice. The potency of nalfurafine in the tests is in the order (high to low) of the acetic acid-induced abdominal constriction test (A_50_ 3.3 μg/kg), mechanical tail pressure (A_50_ 9 μg/kg), tail pinch tests (A_50_ 35 μg/kg), thermal tail flick (A_50_ 62 μg/kg), 51°C hot plate (A_50_ 129 μg/kg). These observations are consistent with the notion that KOR agonists are less potent in thermal nociceptive tests. Coupled with other findings that nalfurafine is highly potent in the formalin test in mice, these observations indicate that nalfurafine, and perhaps other KOR agonists, may be useful in humans for only visceral pain, inflammatory pain and mechanical pain, unlike MOR agonists which appear to be effective for various modalities of acute pain.

Which antinociception tests used also has implications when it comes to examination of dose separation between analgesia and side effects such as CPA, hypolocomotion and motor incoordination. For example, if thermal tests are used for determination of antinociceptive activities of KOR agonists, it is likely that nalfurafine and other KOR agonists would cause undesired side effects within the dose range effective for thermal antinocicpetion.

### 7.4. Dose consideration

Whenever possible, it is best to establish dose-response relationships for both the therapeutic effects and the adverse effects and obtain A_50_ values. It is desirable to get a compound with a large dose separation for the therapeutic vs. adverse effects. For more lengthy or cumbersome experiments, such as CPA, a few doses including doses at the A_50_, A_95_ and 2x A_95_ values of the therapeutic effects should be conducted.

## 8. Conclusion

Studies conducted on mutant mouse lines (GRK3-/-, β-arrestin2-/-, p38alpha MAPK-/-, K4A), distinct KOR agonists and several G protein-biased KOR agonists have consistently demonstrated that analgesic effects and anti-pruritic effects of KOR agonists are mediated by G proteins. On the other hand, studies on the mechanisms underlying KOR-mediated CPA, anhedonia, hypolocomotion, and motor incoordination have yielded conflicting results, particularly CPA. CPA was shown to be mediated by GRK3- and p38alpha MAPK-dependent pathways, to be mediated by the mTOR pathway, or to be downstream of PKC activation, but unrelated to β-arrestin2 or KOR phosphorylation in male mice. On the other hand, KOR phosphorylation is required for CPA to U50,488H in female mice. Several G protein-biased KOR agonists have been synthesized and characterized *in vitro* and *in vivo*. By and large, these G protein-biased KOR agonists produced antinociceptive and/or anti-scratch effects with fewer or lower degrees of side effects in rodents. However, their side effect profiles are not identical and some still produced CPA in rodents. Results from genetic manipulations, pharmacological intervention or different KOR agonists reveal that different mechanisms underlie KOR-mediated CPA, hypolocomotion and motor incoordination. Large-scale phosphoproteomic studies without *a priori* hypothesis have shown that many cellular pathways are modulated by KOR agonists and activation of the mTOR pathway, CB1 receptor and PKC is shown to be involved in CPA to KOR agonists. Phosphoproteomic studies have produced an unprecedent wealth of information, which awaits further analyses to determine their roles in KOR-mediated behaviors. Because the mechanisms underlying adverse effects of KOR agonists are not fully agreed upon, discovery and characterization of KOR agonists for clinical use still require *in vivo* empirical determination of dose separation between beneficial therapeutic effects and adverse side effects. The larger the dose separation, the better the pharmacological profile of the compound.

## Author contributions

L-YL-C wrote and edited the manuscript. PH edited the manuscript. Both authors contributed to the article and approved the submitted version.

## Footnotes

1
https://clinicaltrials.gov/ct2/show/results/NCT03422653
